# Emotions, COVID-19 related thoughts and satisfaction with life during the critical period from control to relaxation

**DOI:** 10.3389/fpsyg.2023.1211614

**Published:** 2023-09-19

**Authors:** Chunting Diao, Huiping Tan, Yanhui Wen, Ruiyue Zhu, Xiaoyue Wu, Shiqi Zhang, Yongzhi Zhao, Nian Liu, Xuan Zhou, Zhen Hu

**Affiliations:** School of Medical Humanities, Hubei University of Chinese Medicine, Wuhan, China

**Keywords:** coronavirus disease 2019 (COVID-19), COVID-19 related thoughts, emotion, satisfaction with life, positive thought, negative emotion

## Abstract

**Introduction:**

In the context of declining mortality rates and increasing infectivity, it has become unavoidable for the majority of individuals to experience a COVID-19 infection at some point. This study aimed to investigate the psychological well-being of the general population during China’s transition period from strict control measures to relaxed policies in COVID-19 prevention and control, as well as the impact of COVID-19 related thoughts on emotion and life satisfaction during widespread infections.

**Methods:**

A cross-sectional study was conducted involving a sample size of 1578 participants. Participants completed self-report questionnaires assessing positive and negative emotions, thoughts about COVID-19, and satisfaction with life. Demographic characteristics such as sex, age, and education level were controlled for in the analysis.

**Results:**

The findings revealed that individuals who had been infected with COVID-19 (specifically the Omicron variant BA.5.2 or BF.7) reported lower levels of positive emotions compared to those who were uninfected or had recovered from the infection. There was a significant relationship between COVID-19-related thoughts, emotions, and life satisfaction. Positive COVID-19 related thoughts were found to mediate the relationship between negative emotions and satisfaction with life.

**Discussion:**

This study represents a comprehensive examination conducted in China, focusing on assessing the impact of the COVID-19 pandemic on the general population during the critical transition period from control to relaxation. Throughout this period, the number of infections experienced fluctuations, initially rising but eventually declining over a one-month span. In such a momentous historical period, maintaining a positive perspective on COVID-19 and its management becomes paramount in enhancing the emotional well-being, life satisfaction and overall well-being of individuals.

## 1. Introduction

The COVID-19 pandemic has become a major global health crisis over the past 3 years, with significant impacts worldwide (Şimşir et al., 2022; Vilca et al., 2022). The Chinese government has responded to the pandemic by implementing strict epidemic prevention and control policies, including measures such as city lockdowns, restrictions on movement, and nationwide quarantines. While these measures have been effective in curbing the spread of the virus, they have also had a significant impact on people’s daily lives. The Chinese government has gradually eased controls after a period of lockdowns and quarantines. On December 7, 2022, the Chinese government issued 10 policies to remove a number of control measures, such as nucleic acid testing and health code verification, to minimize the epidemic’s impact on economic and social development (The Xinhua News Agency, 2022a). On December 26, 2022, according to the comprehensive assessment of the factors such as virus variation, the epidemic situation, China adjusted the novel coronavirus infection from “class B tube A” to “class B tube B.” According to relevant laws and regulations, the quarantine measures for patients infected with COVID-19 will no longer be implemented and close contacts will no longer be identified from January 8, 2023. High and low risk areas are no longer delimited; hierarchical and classified treatment should be implemented for patients infected with COVID-19, and the medical insurance policy should be adjusted in time (The Xinhua News Agency, 2022b). That means China’s three-year epidemic control campaign has ground to a halt. In the month, Chinese people have gone through the change from control to relaxation, and then, at the same time, thousands of people have been affected by the incapacitating effects of COVID-19 (Omicron variant BA.5.2 or BF.7), with severe fever, cough, sore throat, lung infection, weakness and other symptoms. Statistics from the Chinese National Health Commission show that since December 9, 2022, the number of positive cases and the positive rate of nucleic acid testing for SARS-CoV-2 first increased and then decreased. The number of positive cases peaked at 6.94 million on December 22. Severe cases of respiratory failure and sometimes fatal outcomes have occurred, and there has been a massive hospital run.

The COVID-19 pandemic has had a significant negative impact on people’s mental health, leading to a range of psychological distress. Fear and anxiety surrounding the virus have contributed to emotional distress and maladaptive behaviors, such as depression, stress, fear, panic, anger, loneliness, boredom, etc. (Banerjee and Rai, 2020; Esterwood and Saeed, 2020; Özdin and Bayrak Özdin, 2020; Pappa et al., 2020; Pedrosa et al., 2020; Cai et al., 2021; Lindert et al., 2021; Lopez-Leon et al., 2021; Almgren et al., 2022; Balakrishnan et al., 2022). Those with pre-existing mental illnesses and cognitive biases are particularly vulnerable to destabilization (Prochwicz et al., 2020).

Well-being is a multifaceted, ambiguous concept but in general refers to having a positive view of life and circumstances, which equates to being happy and satisfied with life (Diener, 2000; Seckman, 2023). The COVID-19 pandemic has had a profound impact on people’s well-being, mainly due to the uncertainty about the future and the measures governments have implemented to control the virus. These measures, including home confinement, physical distancing, and nationwide lockdowns, have resulted in increased anxiety and fear, as well as a decline in individual satisfaction with life (Lench et al., 2019; Bakioğlu et al., 2021; Esposito et al., 2021; Alimoradi et al., 2022). Additionally, scientific and medical publications, as well as public media reports, have amplified panic, fear, and stigmatization (Luo et al., 2021; Pak et al., 2022). Relaxation of these measures can reduce negative emotions, but life satisfaction may take time to recover (Wang et al., 2020). The pandemic has also led to individuals harboring negative thoughts about the future. University students, for instance, are fearful of the economic, social, and psychological implications on their future careers (Cao et al., 2020; Shindi et al., 2022).

The cognitive processing of stressful events, such as the COVID-19 pandemic, is believed to have a significant impact on individuals’ emotional responses and overall well-being. Lazarus’s (1991) Emotion-Cognition-Well-being Model emphasizes the interplay between emotions, cognition, and well-being. This model suggests that both emotions and cognitive processes significantly impact an individual’s overall well-being. Generally, experiencing positive emotions and engaging in positive cognitive evaluations are associated with higher levels of well-being. On the other hand, negative emotions and negative cognitive evaluations tend to be linked to lower levels of well-being. Additionally, an individual’s cognitive evaluations can influence the generation and regulation of emotions, thereby further shaping their overall well-being (Lazarus, 1991; Lazarus et al., 2010). The meaning-seeking theory posits that individuals actively pursue a sense of meaning and purpose in the face of threatening events. This process enhances their self-efficacy, fosters self-affirmation, and cultivates psychological resilience (Park, 2010). These theories offer a strategy to encourage individuals to proactively deal with the challenges posed by COVID-19 and enhance their emotional well-being and life satisfaction. Studies have demonstrated that individuals have the ability to find positive aspects within difficult situations and learn and grow from them by focusing on their strengths and maintaining a positive perspective (Pyszczynski et al., 2015; Cox et al., 2021; Leibow et al., 2021). It is worth noting that while older people are at a higher risk of infection compared to other age groups and face challenges such as a lack of physical touch, reduced interaction with family, fear of infection, hospitalization, and death, as well as concerns about missing out on active years of life, research suggests that older people may exhibit lower stress levels, higher overall mental well-being, and a better emotional resilience response and coping skills following the experience of COVID-19 compared to younger people (Sterina et al., 2021; Seckman, 2023). Therefore, it is crucial to seek support when needed and engage in self-care practices to promote resilience and well-being.

However, existing literature and reviews mainly focus on measuring anxiety, depression, and stress levels during the COVID-19 pandemic, tending to emphasize the negative consequences of the pandemic’s psychological and psychosocial impact. Limited research has been conducted on the impact of the COVID-19 pandemic on overall well-being (see Esposito et al., 2021; Sterina et al., 2021; Seckman, 2023). Nevertheless, existing research has not explored how people cope with or mitigate the psychological effects of these adverse outcomes, especially during a critical time in the policy transition from control to relaxation, coupled with an increase in the number of infection cases. While previous studies primarily examined the negative impacts of COVID-19, more research is needed to investigate the positive responses to the pandemic and the psychological growth that these responses may bring to individuals. As it becomes more likely that most people will contract the virus, it is essential to explore how individuals maintain a positive outlook and respond to the novel corona virus, as this can have significant implications for their mental health and overall life development.

Building upon previous research and theoretical frameworks, our study aimed to investigate the influence of individuals’ perception of the COVID-19 pandemic on their emotions and life satisfaction. To achieve this, we formulated the following hypotheses:

*H1*: During the crucial phase of transitioning from the control phase to the relaxation phase of the COVID-19 pandemic, there is a significant relationship between individuals’ emotions, thoughts related to COVID-19, and their satisfaction with life.

*H1a*: Positive emotions were positively associated with positive thoughts about the pandemic and higher levels of life satisfaction, while negative emotions were negatively associated with positive thoughts about the pandemic and lower levels of life satisfaction.

*H1b*: Positive thoughts about COVID-19 were positively correlated with satisfaction with life, while negative thoughts were inversely correlated with satisfaction with life.

*H2*: COVID-19 related thoughts acted as mediators in the relationship between negative emotions and satisfaction with life.

*H2a*: Positive thoughts about COVID-19 partially mediate the relationship between positive or negative emotions and satisfaction with life.

*H2b*: Negative thoughts about COVID-19 partially mediate the relationship between positive or negative emotions and satisfaction with life.

## 2. Materials and methods

### 2.1. Study design, participants, and procedure

A cross-sectional study design was employed to investigate the relationships between emotions, COVID-19 related thoughts, and life satisfaction. Data collection was conducted through an online survey method. The survey was hosted on the Wenjuanxing platform, which is an internet-based survey platform. To recruit participants, a questionnaire was created on Wenjuanxing and QR code images as well as survey links were generated. Participants were recruited from China through internet forums and popular social media platforms such as WeChat and QQ. The recruitment period was from December 13th, 2022, to January 13th, 2023.

We actively promoted and shared the questionnaire links with potential participants through social media platforms such as WeChat and QQ, both through group chats and individual conversations. Additionally, we utilized the wide reach of social media platforms, such as WeChat Moments, to post the survey links and QR codes. We also requested friends, family, colleagues, and others to help forward and share them, in order to attract more potential participants. Individuals could access the online web survey by scanning QR codes posted on WeChat or QQ using their mobile phones or by clicking on the provided survey link. A total of 1,610 individuals completed our questionnaire.

The study inclusion criteria were as follows: (1) individuals aged 18 years or older who were capable of independently completing an online questionnaire; (2) those in good health with no history of COVID-19 infection, major illness, or surgery before the Omicron variant BA.5.2 or BF.7 epidemic in December 2022. Participants were asked to self-report any mental health conditions in the questionnaire, and those who reported pre-existing mental health conditions or were receiving treatment for depression or anxiety were excluded. However, it should be noted that due to the self-reported nature of mental health conditions in the questionnaire, there is a possibility that individuals with undiagnosed pre-existing conditions could meet the survey inclusion criteria. This limitation is challenging for us to avoid. Additionally, the exclusion criteria were as follows: (1) responses completed in less than 60 s; (2) participants whose responses displayed a high degree of consistency with the same answer throughout the questionnaire. Among the participants who accessed the survey, 98.01% met these inclusion criteria, resulting in a final sample size of 1,578.

Before the formal survey, a preliminary question was included to inquire whether participants agreed to participate in the study. Only those who agreed were guided to proceed with answering the subsequent survey questions. If participants did not agree, they were unable to continue with the survey. All participants were informed that their responses would be anonymous, and participation was voluntary. Participants provided consent to participate, and prior to data collection, the study was approved by the Institutional Ethics Committee of the School of Humanities at Hubei University of Chinese Medicine (ethical clearance number: P_2022037) to ensure the ethical treatment of human participants.

### 2.2. Measures

#### 2.2.1. Demographics

Self-reported data were collected for socio-demographic factors such as sex, age, education and COVID-19 infection status.

#### 2.2.2. Measures of emotions

Emotional measures were obtained using the 10-item short form of the Positive and Negative Affect Schedule (Mackinnon et al., 1999). This scale assesses both positive and negative emotions, with 5 items for each. The 5 positive emotion items were inspired, alert, excited, enthusiastic, and determined, while the 5 negative emotion items were afraid, upset, nervous, scared, and distressed. Participants rated the extent to which they had felt each emotion in the past month on a scale ranging from 1 (not at all) to 5 (extremely). Mean scores for positive and negative affect were calculated based on the individual responses to all 5 items on each scale. The maximum possible score was 5 and the minimum possible score was 1 for both positive and negative affect. The short-form version of the PANAS has previously been used as a reliable and valid tool for dynamic affective processes and demonstrated good internal consistency and reliability (Thompson, 2007; Cooke et al., 2022). The reliabilities of the scale which measured in Chinese students by Cronbach’s alpha were 0.81 for positive affect and 0.83 for negative affect, revealing excellent internal consistency reliability (Liu et al., 2020). The present study yielded high Cronbach’s alpha values for positive (0.78) and negative (0.89) emotions, indicating good reliability.

#### 2.2.3. Measures of thoughts

To measure thoughts, a self-developed questionnaire comprising of 10 questions in two sections was used. The construction of these 10 questions was inspired by existing literature in the field (Segerstrom, 2005; Brooks et al., 2020). Prior to administering the questionnaire, a pretesting process was conducted to improve internal consistency and scale reliability. This involved pilot testing the questionnaire with a small sample of participants and making necessary adjustments based on their feedback.

The first section consisted of 5 questions on positive thoughts, while the second section had 5 questions on negative thoughts. Participants rated each item on a 6-point scale ranging from 1 (not at all) to 6 (very much). Principal Component Analysis (PCA) with varimax rotation was employed to analyze the data and explore the underlying factor structure. An item retention heuristic was applied, and any items that did not load above 0.40 were excluded from the analysis. This procedure resulted in a simple factor structure, as depicted in Table 1. All 10 items demonstrated significant loadings onto an unrotated PCA factor at a level higher than 0.40. The two factors identified accounted for a substantial portion of the variance. The final 10-item two-factor solution, along with the factor loadings, is presented in Table 1.

**Table 1:** Item loading.

Item	Positive thoughts	Negative thoughts
The epidemic is terrible.		0.729
I am afraid of being infected.		0.788
I think this epidemic is difficult to end in a short time.		0.731
I believe that with your efforts, the epidemic will soon pass.	0.759	
I think the epidemic is spreading too fast to contain.		0.750
No matter how the epidemic develops, we can control it.	0.800	
I’m ready to fight the epidemic for a long time.	0.634	
I am extremely confident that we can successfully contain the epidemic.	0.890	
I believe in official reports more than rumors.	0.792	
Many official reports are lagging behind, and some rumors are true.		0.583

The Measure of Thoughts demonstrated good internal consistency, as indicated by a Cronbach’s alpha value of 0.72 for the overall scale. Additionally, both factors showed reliable internal consistency, with Cronbach’s alpha values of 0.84 for positive thoughts and 0.75 for negative thoughts.

#### 2.2.4. Satisfaction with life

The satisfaction with life scale developed by Diener et al. (1985) is a subjective measure and reflects the individual’s own evaluation of their life, which was utilized to assess the participants’ satisfaction with life in this study. The scale contains 5 items, such as “The conditions of my life are excellent.” Participants respond to the 5 items on a 7-point scale ranging from 1 (“Strongly Disagree”) to 7 (“Strongly Agree”). The scale has been heavily used as a reliable and valid tool for assessing the life satisfaction component of subjective well-being, and it is sensitive to changes in life satisfaction due to various factors like life events and therapy (Pavot and Diener, 2008). The satisfaction with life scale has demonstrated good test–retest stability, the mean Cronbach’s alpha across 76 reliability coefficients in 62 articles utilizing the scale was 0.78 with a 95% confidence internal ranging from 0.766 to 0.807 (Vassar, 2008). We chose to use satisfaction of life as a measure of well-being for several reasons. Firstly, satisfaction of life is widely accepted and validated as a measure of well-being, as supported by various studies (Levacher et al., 2023). Secondly, the Satisfaction with Life Scale is a subjective measure that reflects individuals’ own evaluation of their life. Assessing the quality of life through individuals’ subjective experiences is a common approach to measure well-being (Pavot and Diener, 2008). Lastly, satisfaction of life is relatively easy to measure and understand, making it accessible to a wider range of participants (Vassar, 2008). While there are other measures of well-being available, given our aim to promptly assess the impact of pandemic developments on individuals’ subjective feelings, satisfaction of life was seen as a suitable option for our study. In this study, the satisfaction with life measure showed high internal consistency reliability, with a Cronbach’s alpha coefficient of 0.92.

#### 2.2.5. Control variables

Sex, age, education level, and COVID-19 infection status were included as control variables, as these variables may interfere with or affect the relationships between other variables in the model. For instance, previous research has shown that older age, lower education level may be associated with lower life satisfaction (Levacher et al., 2023). Additionally, controlling sex is important as there may be gender differences in emotion regulation, which could also affect the relationship between emotions, positive thoughts, and satisfaction with life (Bartels, 2015; Liu et al., 2020).

### 2.3. Statistical analysis

The statistical software SPSS 20 (IBM, New York, NY, USA) was used to conduct data analysis in this study. The dataset was examined for improbability, normality, univariate outliers, and missingness. Categorical data were expressed in frequency and percentage, whereas continuous data were expressed as the mean ± SD for normally distributed data. To analyze the association relationship, appropriate statistical tests such as independent samples *t*-test, one-way analysis of variance (ANOVA), and correlation analyses (Pearson’s) were to assess the predicted relationships between positive emotion, negative emotion, positive thoughts, negative thoughts, and satisfaction with life. Pearson coefficients range from +1 to −1, indicating a positive or negative correlation respectively, while 0 represents no correlation.

To investigate whether positive thoughts and negative thoughts mediate the relationship between positive/negative emotion and satisfaction with life, the researchers utilized the PROCESS macro (model 4) developed by Hayes (2018). This analysis was conducted while controlling for sex, age, education level, and COVID-19 infection status. The PROCESS macro estimates the significance of the cross-product of path A, which is the mediator-predictor coefficient, and path B, the outcome-mediator coefficient, while controlling for the predictor. To test the mediation effect, we employed Hayes’s method (Hayes, 2018) with 5,000 bootstrapped samples to obtain the 95% bias-corrected and accelerated confidence intervals (CIs) of the indirect effects of positive and negative emotions on life satisfaction through positive and negative thoughts, respectively. If the CI of the indirect effect did not include zero, a significant mediation effect was concluded. All statistical analyses were conducted at a 95% confidence interval, and *p* < 0.05 was considered statistically significant.

## 3. Results

### 3.1. Characteristics of participants

Among the 1,578 participants who enrolled in this study, the majority (*n* = 1,329, 84.2%) were aged 30 or below at the start of the study. Female participants (*n* = 996, 63.1%) slightly outnumbered their male counterparts. The majority of participants held a graduate-level education (*n* = 1,245, 78.9%). Most of the participants were dealing with the Omicron variant BA.5.2 and BF.7 of COVID-19. Most have been recovered (*n* = 1,089, 69.0%). The factors analyzed are significant difference between categorical variables (Table 2).

**Table 2:** Characteristics of participants (*n* = 1,578).

Characteristic		Frequency (%)
Sex	Male	582 (36.9)
	Female	996 (63.1)
Age (years)	≤30	1,329 (84.2)
	31–40	108 (6.8)
	41–50	72 (4.6)
	51–60	60 (3.8)
	>60	9 (0.6)
Educational level	Secondary school	135 (8.6)
	Graduate	1,245 (78.9)
	Postgraduate	198 (12.5)
COVID-19 infection status (Omicron variant BA.5.2 or BF.7)	Uninfected	426 (27.0)
	Infecting	63 (4.0)
	Recovered	1,089 (69.0)

### 3.2. Comparing differences in demographic variables

Significant differences in positive emotions were found between the uninfected, infected and recovered groups. The participants who were infected COVID-19 (Omicron variant BA.5.2 or BF.7) have the lower positive emotion compared to the uninfected group and the recovered group. However, no significant differences were found in positive emotion between sex, age and educational level. In contrast, there were no statistically significant differences found in negative emotions related to any demographic characteristics of the participants (Table 3).

**Table 3:** *T*-test and ANOVA analysis of the variables with characteristics of participants.

Characteristic	Positive emotion	Negative emotion	Positive thoughts	Negative thoughts	Satisfaction with life
Sex					
Male	14.55 ± 3.47	11.93 ± 4.20	21.46 ± 5.96	18.06 ± 5.18	20.18 ± 7.43
Female	14.19 ± 3.49	12.20 ± 4.63	23.05 ± 5.08	19.35 ± 5.02	19.94 ± 7.12
*t*	1.12	−0.67	−3.23**	−2.81**	0.38
Age (years)					
≤30	14.25 ± 3.44	12.01 ± 4.37	22.27 ± 5.38	18.54 ± 4.97	19.74 ± 7.08
31–40	14.38 ± 3.56	13.55 ± 4.83	23.69 ± 5.64	21.47 ± 5.04	20.17 ± 8.40
41–50	14.50 ± 3.86	12.04 ± 5.24	20.92 ± 6.54	20.87 ± 6.20	20.71 ± 6.80
51–60	15.60 ± 4.06	11.45 ± 4.61	25.65 ± 4.54	19.40 ± 5.34	24.55 ± 7.26
>60	14.33 ± 2.31	13.67 ± 7.77	27.67 ± 2.08	17.67 ± 4.93	25.33 ± 8.74
*F*	0.74	1.2	3.51** 4>3; 4>1; 5>3	3.90** 2>1; 3>1	2.63* 4>1; 4>2
Educational level					
1. Secondary school	14.00 ± 4.17	10.08 ± 4.68	23.42 ± 6.74	17.20 ± 5.20	21.44 ± 8.71
2. Graduate	14.32 ± 3.34	12.17 ± 4.35	22.44 ± 5.28	18.99 ± 5.02	19.56 ± 6.89
3. Postgraduate	14.46 ± 3.86	12.56 ± 4.98	21.95 ± 5.70	19.30 ± 5.49	22.05 ± 7.80
*F*	0.35	2.31	0.98	2.76* 3>2>1	4.37* 3>2
COVID-19 infection status (Omicron variant BA.5.2 or BF.7)					
Uninfected	14.50 ± 3.52	12.01 ± 4.49	23.01 ± 5.57	18.84 ± 5.13	19.80 ± 7.27
Infecting	11.95 ± 4.09	14.00 ± 5.41	18.52 ± 4.74	20.18 ± 6.80	17.14 ± 8.82
Recovered	14.22 ± 3.17	12.06 ± 4.24	21.65 ± 4.99	18.68 ± 4.76	21.05 ± 6.72
*F*	5.47** 1>2; 3>2	1.98	9.06*** 1>3>2	1.62	3.30* 3>2

Sex, age, and COVID-19 infection status were found to be significantly (*p* < 0.01) associated with positive thoughts. Specifically, females and uninfected participants had higher scores on positive thoughts. The groups of participants aged 51–60 years old and over 60 years old (whose scores were similarly high) had higher scores than the groups aged 41–50 years old and under 30 years old (whose scores were similarly low). The detailed correlation results are presented in Table 3.

Sex, age, and educational level were found to be significantly (*p* < 0.01) associated with negative thoughts, with lower scores on males and the group of secondary educational level. The group of participants under 30 years old had significantly lower scores than the groups aged 31–40 years old and 41–50 years old (whose scores were similarly low). The detailed correlation results are presented in Table 3.

Age, educational level, and COVID-19 infection status were found to be significant predictors (*p* < 0.01) of satisfaction with life. The results showed that older participants (51–60 years old) had higher satisfaction scores than the group of participants aged 31–40 years old and the group of participants under 30 years old (whose scores were similar). Postgraduate students were more satisfied than undergraduates. The participants who recovered from the COVID-19 (Omicron variant BA.5.2 or BF.7) had higher scores the infecting ones (Table 3).

### 3.3. Descriptive statistics and correlation analysis

The observed variables’ means, standard deviations, and correlations were displayed in Table 4. The means (SD) of positive emotion, negative emotion, positive thoughts, negative thoughts, and satisfaction with life were 14.32 (3.48), 12.10 (4.47), 22.46 (5.47), 18.87 (5.11), and 20.03 (7.23), respectively. Correlation analyses were conducted to examine the relationships between the variables. These analyses revealed several significant findings. Firstly, positive emotion showed a positive correlated with positive thoughts (*r* = 0.33, *p* < 0.01) and satisfaction with life (*r* = 0.49, *p* < 0.01). This suggested that individuals experiencing higher levels of positive emotion also reported more positive thoughts and greater satisfaction with life. Secondly, negative emotion exhibited a positive correlation with negative thoughts (*r* = 0.39, *p* < 0.01). Additionally, it was inversely correlated with positive thoughts (*r* = −0.17, *p* < 0.01) and satisfaction with life (*r* = −0.20, *p* < 0.01). These findings indicated that higher levels of negative emotion were associated with more negative thoughts and lower levels of both positive thoughts and satisfaction with life. Lastly, positive thoughts displayed a moderate positive correlation with satisfaction with life (*r* = 0.43, *p* < 0.01). This suggested that individuals who reported more positive thoughts also tended to have a higher level of satisfaction with life.

**Table 4:** Pearson correlations between the main study variables.

Variables	*M*	SD	1	2	3	4	5
Positive emotion	14.32	3.48	1				
Negative emotion	12.10	4.47	0.08	1			
Positive thoughts	22.46	5.47	0.33**	−0.17**	1		
Negative thoughts	18.87	5.11	−0.05	0.39**	0.03	1	
Satisfaction with life	20.03	7.23	0.49**	−0.20**	0.43**	−0.05	1

### 3.4. Mediation analyses

To investigate the potential mediating role of thoughts, mediation analyses were conducted, controlling for sex, age, education level, and COVID-19 infection status. Previous research suggests that sex, age, and COVID-19 infection status may influence cognition and life satisfaction, so we included them as covariates in our analysis (Bartels, 2015; Liu et al., 2020; Levacher et al., 2023). The results were presented below, with a focus on enhancing clarity and ensuring all findings were appropriately discussed.

#### 3.4.1. Positive thoughts as mediator between positive emotions and life satisfaction

**Figure 1:**
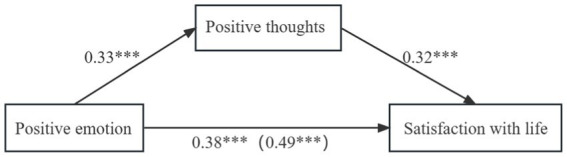
The mediation effect of positive thoughts on the association between positive emotion and satisfaction with life. ***p < 0.001.

Table 5 and Figure 1 depicted the results, showing that the completely standardized indirect effect of the path mediated by positive thoughts was 0.10 (95% CI = [0.07, 0.14]). The confidence intervals of the indirect effect did not include zero, indicating that positive thoughts partially mediated the relationship between positive emotions and life satisfaction. Specifically, the relative mediating effect of positive thoughts was estimated to be 21.55%.

**Table 5:** The mediation effect of positive thoughts on the relationship between positive emotions and life satisfaction.

	Satisfaction with life		Positive thoughts		Satisfaction with life	
	Model 1		Model 2		Model 3	
	*β*	SE	*β*	SE	*β*	SE
Sex	−0.01	0.57	0.16***	0.46	−0.06	0.55
Age	0.11**	0.36	0.06	0.29	0.09*	0.34
Educational level	0.04	0.61	−0.04	0.49	0.05	0.58
COVID-19 infection status	0.09**	0.31	−0.12**	0.25	0.13***	0.30
Positive emotion	0.49***	0.08	0.33***	0.06	0.38***	0.08
Positive thoughts					0.32***	0.05
*R*	0.51		0.39		0.59	
*R^2^*	0.26		0.15		0.35	
Residual standard error	38.95		25.59		34.55	
*F* statistic	*F*(5, 1,572) = 36.81***		*F*(5, 1,572) =18.92***		*F*(6, 1,571) =45.80***	

*p < 0.05, **p < 0.01, ***p < 0.001, shaded variables were standardized.

**Figure 2:**
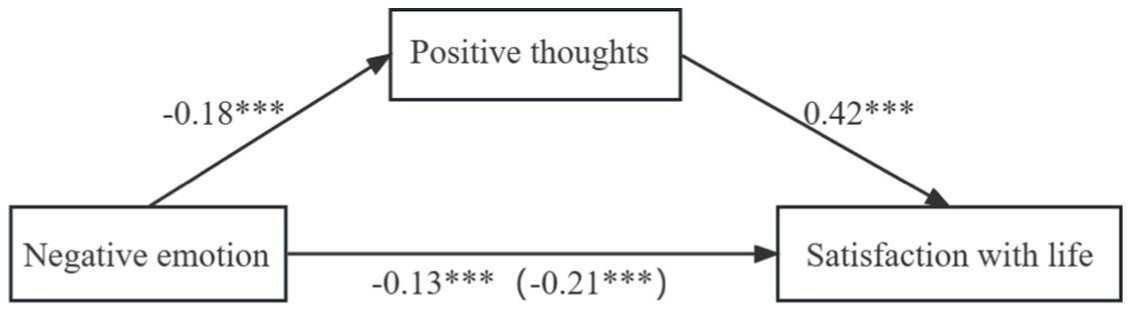
The mediation effect of positive thoughts on the association between negative emotion and satisfaction with life. ***p < 0.001.

Similarly, as shown in Table 6 and Figure 2, the completely standardized indirect effect of the path mediated by positive thoughts was −0.08 (95% CI = [−0.12, −0.03]). Again, the confidence intervals of the indirect effect did not contain zero, suggesting that positive thoughts also had a partial mediation effect on the association between negative emotion and satisfaction with life. The relative mediation effect was estimated to be 36.00%.

**Table 6:** The mediation effect of positive thoughts on the relationship between negative emotions and life satisfaction.

	Satisfaction with life		Positive thoughts		Satisfaction with life	
	Model 1		Model 2		Model 3	
	*β*	SE	*β*	SE	*β*	SE
Sex	−0.03	0.64	0.16***	0.48	−0.06*	0.59
Age	0.15***	0.40	0.09*	0.31	0.11**	0.37
Educational level	0.08	0.69	−0.01	0.49	0.08	0.62
COVID-19 infection status	0.07	0.35	−0.13**	0.26	0.13***	0.32
Negative emotions	−0.21***	0.07	−0.18***	0.05	−0.13***	0.06
Positive thoughts					0.42***	0.05
*R*	0.26		0.28		0.48	
*R^2^*	0.07		0.08		0.23	
Residual standard error	49.14		27.90		40.49	
*F* statistic	*F*(5, 1,572) = 7.62***		*F*(5, 1,572) =8.76***		*F*(6, 1,571) =26.37***	

*p < 0.05, **p < 0.01, ***p < 0.001, shaded variables were standardized.

#### 3.4.2. Negative thoughts as mediator between negative emotions and life satisfaction

Furthermore, we explored the potential mediation effect of negative thoughts on the relationship between positive/negative emotions and life satisfaction. The results are presented in Tables 7, 8.

**Table 7:** The mediation effect of negative thoughts on the relationship between positive emotions and life satisfaction.

	Satisfaction with life		Negative thoughts		Satisfaction with life	
	Model 1		Model 2		Model 3	
	*β*	SE	*β*	SE	*β*	SE
Sex	−0.01	0.57	0.12*	0.46	−0.01	0.57
Age	0.11**	0.36	0.12*	0.29	0.11**	0.36
Educational level	0.04	0.61	−0.12*	0.49	0.04	0.62
COVID-19 infection status	0.09*	0.31	−0.03	0.25	0.09*	0.31
Positive emotions	0.49***	0.08	−0.05	0.06	0.49***	0.08
Negative thoughts					−0.04	0.05
*R*	0.51		0.20		0.51	
*R^2^*	0.26		0.04		0.26	
Residual standard error	38.95		25.36		38.96	
*F* statistic	*F*(5, 1,572) = 36.81***		*F*(5, 1,572) =4.19**		*F*(6, 1,571) =30.83***	

**Table 8:** The mediation effect of negative thoughts on the relationship between negative emotions and life satisfaction.

	Satisfaction with life		Negative thoughts		Satisfaction with life	
	Model 1		Model 2		Model 3	
	*β*	SE	*β*	SE	*β*	SE
Sex	−0.03	0.64	0.11**	0.42	−0.03	0.65
Age	0.15***	0.41	0.10*	0.27	0.15***	0.41
Educational level	0.08	0.69	0.08	0.46	0.08	0.69
COVID-19 infection status	0.07	0.35	−0.03	0.23	0.07	0.35
Negative emotions	−0.21***	0.07	0.38***	0.5	−0.21***	0.07
Negative thoughts					0.02	0.07
*R*	0.26		0.43		0.26	
*R^2^*	0.07		0.18		0.07	
Residual standard error	49.14		21.60		49.22	
*F* statistic	*F*(5, 1,572) = 7.62***		*F*(5, 1,572) =23.01***		*F*(6, 1,571) =6.37***	

The completely standardized indirect effect of the path mediated by negative thoughts on the association between positive emotion and satisfaction with life was 0.002 (95% CI = [−0.004, 0.013]); and on the association between negative emotion and satisfaction with life was 0.01 (95% CI = [−0.03, 0.05]). Both confidence intervals for indirect effects contain zero, indicating that negative thoughts did not significantly mediate the relationship between positive/negative emotions and life satisfaction.

Overall, our findings suggest that positive thoughts partially mediate the relationship between positive/negative emotions and life satisfaction. However, negative thoughts did not have a significant mediating effect on this relationship. These results highlight the importance of positive thoughts in enhancing life satisfaction, particularly in the context of negative emotions.

## 4. Discussion

The aim of our study was to investigate the relationships between individuals’ thoughts about the COVID-19 pandemic, their emotions and their overall satisfaction with life. Additionally, we explored whether thoughts about COVID-19 mediate the association between emotions and satisfaction with life during a time of widespread infection, as China transitions from strict control to relaxed policies within COVID-19 prevention and control measures.

Our results revealed a significant relationship between individuals’ thoughts about the pandemic and their emotional experiences and satisfaction with life, which supporting Hypothesis 1. Specifically, we found that positive emotions were positively associated with positive thoughts about the pandemic and higher levels of life satisfaction. On the other hand, negative emotions were positively associated with negative thoughts and lower levels of life satisfaction. This finding Supported Hypothesis 1a. Additionally, we observed a positive correlation between positive thoughts about COVID-19 and satisfaction with life, and a negative correlation between negative thoughts about COVID-19 and satisfaction with life, thus supporting Hypothesis 1b.

Furthermore, through conducting moderated mediation analyses, we found that positive thoughts about COVID-19 mediated the relationship between positive/negative emotions and the satisfaction with life. However, we did not find any evidence of mediation effects for negative thoughts about COVID-19. This partially supports Hypotheses 2 and 2a, but does not support Hypothesis 2b. This suggests that positive thoughts about the pandemic play a role in explaining the link between emotions and satisfaction with life, whereas negative thoughts do not appear to have a significant mediating effect.

### 4.1. The selection of variables, research design, and the importance of the study

As previously mentioned, well-being is a complex and multifaceted concept that generally refers to having a positive outlook on life and being happy and satisfied (Diener, 2000; Seckman, 2023). It can be categorized into three types: psychological well-being, social well-being and emotional well-being (Snyder and Lopez, 2007). In this study, we specifically focused on emotional well-being, which refers to life satisfaction, positive emotions, while minimizing negative emotions. For measuring these aspects, we have chosen the Satisfaction with Life Scale (SWLS) and the Positive and Negative Affect Schedule (PANAS), both widely used and validated measurement tools (Cooke et al., 2022; Levacher et al., 2023).

The context of our study focused on the rapid transition of the Chinese government’s COVID-19 control policy, which shifted from strict regulations to a state of total openness. This sudden change, coupled with subsequent widespread infections, caught many people off guard. In order to quickly assess the impact of COVID-19 on individuals’ emotions, cognition, and life satisfaction, we opted for a cross-sectional design. This design allowed us to efficiently gather a substantial amount of data and obtain relevant information in a timely manner. While alternative methods, such as a longitudinal design encompassing multiple time points, may provide insights into the long-term effects and dynamic associations between variables, it was our primary objective to capture the immediate impact of this unprecedented historical moment on people’s well-being. We highly valued the opportunity to examine this unique situation and its implications for individuals’ overall well-being. Through the utilizing of a cross-sectional design, we were able to promptly capture this historical moment, providing a snapshot of the pandemic’s impact situation and generating hypotheses and guidance for future research. While the COVID-19 pandemic has had a profound impact on the mental health of individuals, leading to the emergence of a variety of mental illnesses (see Alimoradi et al., 2022; Şimşir et al., 2022), we specifically selected participants without pre-existing mental health conditions to better understand how COVID-19 has affected the well-being of ordinary, healthy individuals.

Improving life satisfaction can enhance well-being, with positive emotions often serving as indicators of well-being. However, it’s important to acknowledge that various stressors and the negative emotions they bring about do not promote well-being (Esposito et al., 2021). The COVID-19 pandemic has had a profound impact on individuals’ sense of well-being, leading to increased stress levels and negative emotions. Existing literature and reviews have primarily focused on measuring anxiety, depression, and stress levels during the pandemic (Lindert et al., 2021; Alimoradi et al., 2022; Almgren et al., 2022; Şimşir et al., 2022), while paying limited attention to the overall well-being of individuals (see Esposito et al., 2021; Sterina et al., 2021; Seckman, 2023). Consequently, the exploration of the relationship between the COVID-19 pandemic and well-being is crucial for understanding its impact and designing effective interventions to support overall well-being.

### 4.2. Controlled variables and their effects on variables

In order to understand the factors that influence well-being during difficult times, it is essential to control some individual and societal factors such as health, age, financial status, job satisfaction and so on (Pappa et al., 2020; Bakioğlu et al., 2021; Lopez-Leon et al., 2021). In our study, we also took into account the variables of sex, age, education level, and COVID-19 infection status. Our findings showed that age, education level, and COVID-19 infection status were significant predictors of life satisfaction (see Table 3), aligning with previous research findings (Pappa et al., 2020; Cox et al., 2021; Balakrishnan et al., 2022). However, we did not consider economic factors in our study due to the specific context of our research period, which was conducted from December 13th, 2022, to January 13th, 2023, during a time when the social and economic situation in China differed from the initial lockdown period in 2020. It was observed that individuals’ economic incomes were not significantly affected during this time. Additionally, to account for the potential influence of mental health status, particularly mental illnesses, on subjective well-being, we specifically controlled for participants’ mental health status. None of the participants reported a history of mental illness, such as anxiety or depression. By controlling for mental health status, our aim was to gain a clearer understanding of the impact of COVID-19 on subjective well-being.

Our analysis revealed that participants who were infected with COVID-19 (specifically Omicron variant BA.5.2 or BF.7) had lower levels of positive emotions compared to the uninfected group and the group that had recovered from the infection. Furthermore, uninfected participants displayed more positive thoughts about the COVID-19 pandemic than infected participants. Additionally, those who had recovered from COVID-19 exhibited higher satisfaction with life scores compared to those currently infected, as shown in Table 3. Furthermore, sex, age was found to have a significant association (*p*s < 0.01) with positive or negative thoughts.

These findings suggest that contracting COVID-19 may evoke negative emotions and reduce life satisfaction. However, the satisfaction with life of individuals who have recovered from the infection tends to return to normal or even surpass the levels of those who have never been infected. This indicates that the negative effects on mental health caused by COVID-19 are not necessarily permanent, and individuals can recover and even experience increased satisfaction with their lives after overcoming setbacks.

### 4.3. Relationship between emotions, COVID-19 related thoughts, and satisfaction with life

Our study found a significant relationship between individuals’ thoughts about the COVID-19 pandemic and their emotions, supporting our hypothesis (H1a). This finding was consistent with prior research on the connection between cognitive factors and emotional outcomes (e.g., Prochwicz et al., 2020). Furthermore, our results suggested that this relationship is stronger during periods of widespread infection when COVID-19 prevention and control measures were being relaxed, compared to periods of strict control. One possible explanation for this finding is that individuals may feel less in control and more vulnerable during a time of relaxed policies, which could amplify the impact of their thoughts about COVID-19 on their emotions (e.g., Lench et al., 2019; Esterwood and Saeed, 2020; Bakioğlu et al., 2021).

Our study also discovered a significant correlation between positive thoughts about COVID-19 and life satisfaction, while negative thoughts were inversely related to life satisfaction, supporting H1b. This finding suggested that the way individuals perceive and think about the COVID-19 pandemic during this unprecedented period may play an important role in their subjective well-being. Our findings aligned with previous research on the role of cognitive factors in subjective well-being (e.g., Cox et al., 2021; Levacher et al., 2023). The fact that positive thoughts were associated with greater life satisfaction reinforces the idea that positive thoughts could be a powerful tool for improving well-being, even in the face of stressful life events. However, it was important to note that our study was not able to establish a causal relationship between thoughts about COVID-19 and life satisfaction. It was plausible that individuals who naturally possessed more optimism or resilience were more likely to have positive thoughts about the pandemic and higher levels of life satisfaction, rather than the reverse scenario.

People’s quality of life is influenced by the satisfaction of various human needs, including the presence of positive thoughts and emotions (Sirgy, 2021). In addition to the direct impact of the COVID-19 pandemic itself, individuals’ thought patterns and emotional states play a significant role in shaping their well-being. Emotions are an individual’s response to external, objective circumstances, while thoughts are subjective and influenced by temperament, personality, values, goals, and cultural and societal factors. Emotions can impact cognition, and in turn, cognition can influence one’s subjective feelings. When faced with similar changes in the external environment, some individuals adapt better than others, which could be influenced by their perceptions and understanding of the COVID-19 pandemic. Our findings suggested that individuals’ positive thoughts about the COVID-19 pandemic and their overall positive emotional state contribute significantly to their overall well-being.

### 4.4. Positive thoughts acted as mediators in the relationship between emotions and satisfaction with life

By controlling sex, age, education level and COVID-19 infection status, we can better isolate the effects of the variables of interest (i.e., positive/negative emotions experienced by individuals and positive thoughts) on life satisfaction and determine whether positive thoughts mediate the relationship between emotions and life satisfaction with higher validity and reliability. The results of our study suggested that positive thoughts about COVID-19 play an important mediating role in the relationship between positive or negative emotions and satisfaction with life (H2a). Our findings indicated that individuals who maintain positive thoughts about COVID-19 were more likely to experience greater subjective well-being, even in the face of challenging circumstances. Interestingly, we did not find evidence that negative thoughts about COVID-19 had mediating effects. This suggests that negative thoughts about the pandemic may not be as important for determining levels of life satisfaction as positive thoughts. Previous studies have found that infection with COVID-19 can have a negative impact on an individual’s cognition for a considerable period of time (Lindert et al., 2021; Lopez-Leon et al., 2021; Almgren et al., 2022; Balakrishnan et al., 2022; Şimşir et al., 2022; Vilca et al., 2022). This could be related to the extent of damage to people’s bodies caused by the novel coronavirus at the time. It is possible that negative thoughts may have a more immediate impact on emotional states, but positive thoughts may have a more enduring influence on overall well-being. As the infectivity of novel coronavirus increases, the disease-causing symptoms are weakened and the impact on the individual’s physical damage is reduced, but the impact on the individual’s mental health is not as severe. In this context, how individuals view and respond positively to COVID-19 infection is critical to their mental health. While heightened mortality awareness may cause an increase in negativity and defensiveness (Schippers and Ziegler, 2019), it is important to note that increased fear and anxiety can also contribute to post-traumatic growth and finding meaning in difficult experiences (Pyszczynski et al., 2015; De Jong et al., 2020). For instance, individuals who report greater existential concerns related to COVID-19 may experience greater emotional and psychological well-being (Cox et al., 2021). Therefore, we do not need to blindly ask people to reduce negative perceptions, which are unavoidable in the role of negative emotions. But what we can do to retain or build as many positive perceptions as possible is the key to later life satisfaction.

Our study also emphasized the importance of exploring potential moderators in relation to well-being. Previous research has demonstrated that personality traits like optimism and mastery resilience may moderate the impact of stressful events on mental health outcomes (e.g., Park, 2010; De Jong et al., 2020; Esterwood and Saeed, 2020). Still, it is important to acknowledge that our study cannot establish a causal relationship between COVID-19 perception and life satisfaction. It is plausible that individuals with inherent optimism or effective coping mechanisms exhibit greater resilience, making them more likely to hold positive thoughts about the pandemic and experience higher levels of life satisfaction, as supported by other studies (e.g., Sterina et al., 2021; Seckman, 2023). Furthermore, it has been suggested that frustration serves as the foundation for psychological resilience (Snyder, 2021). Although our study alone cannot provide sufficient evidence, it is theoretically possible that individuals may have developed increased mental resilience as a result of experiencing the pandemic for 3 years. It would be interesting to explore whether these personality factors moderate the mediating effects of positive thoughts about COVID-19 on satisfaction with life. Nonetheless, these findings had important implications for public health interventions to mitigate the psychological impact of infectious disease outbreaks. Interventions that focus on promoting positive thinking and reducing negative thoughts about the pandemic may be effective in promoting greater satisfaction with life among individuals who are struggling to adjust to this new reality. Overall, our study provided valuable insights into the complex relationship between cognitive factors, emotions, and well-being during the COVID-19 pandemic, and had practical implications for promoting well-being. Promoting positive thinking about the pandemic may be key to improving overall life satisfaction, especially among those who were finding it difficult to cope with the current situation. Continued research is needed to explore these relationships, as well as potential moderators and mechanisms underlying these effects.

### 4.5. Limitations and future directions

Unfortunately, our study did not focus on the dynamical changes in people’s metal health during this period. Future studies could consider longitudinal designs to track the psychological changes of individuals during this critical historical period. Additionally, future research could further explore strategies to help people develop and maintain positive thoughts during public health and security emergencies or other real-world catastrophes. It is important to investigate these factors in greater depth, along with other potential moderators of the relationship between thoughts about COVID-19 and satisfaction with life, to better understand how to promote resilience and well-being during global crises.

## 5. Conclusion

Despite the negative emotional experiences brought by the COVID-19 pandemic, an individual’s cognitive appraisal can significantly affect their subjective experience. The analysis showed a significant correlation between an individual’s thoughts about the pandemic, their emotions, and their overall life satisfaction. The participants who were infected COVID-19 (specifically the Omicron variant BA.5.2 or BF.7) reported lower levels of positive emotion compared to both the uninfected group and the group of recovered individuals. Positive thoughts related to COVID-19 were found to mediate the relationship between negative emotions and life satisfaction. An individual’s perception of COVID-19 can have a significant impact on their emotion and life satisfaction, which may persist for extended periods of time. Fostering a positive cognitive outlook is crucial in helping individuals navigate this sudden and unprecedented crisis during China’s transition from strict to relaxed policies in COVID-19 prevention and control measures. Therefore, it is essential to help individuals build positive perceptions to alleviate negative emotions and improve overall life satisfaction.

## Data availability statement

The original contributions presented in the study are included in the article/supplementary material, further inquiries can be directed to the corresponding authors.

## Ethics statement

The studies involving humans were approved by the Institutional Ethics Committee of the School of Humanities at Hubei University of Chinese Medicine. The studies were conducted in accordance with the local legislation and institutional requirements. The ethics committee/institutional review board waived the requirement of written informed consent for participation from the participants or the participants’ legal guardians/next of kin because the online nature of data collection. A single question was included in the survey, asking participants whether they consent to participate. If participants agreed, they were able to proceed with the survey, and if not, they could choose to discontinue. It has been clearly stated to participants that the survey is anonymous, and the risks associated with this questionnaire are minimal.

## Author contributions

CD: conceptualization, supervision, review, and editing. CD, HT, YW, and RZ: methodology. NL: formal analysis. HT, YW, and RZ: investigation. XW, SZ, ZH, and YZ: resources. CD and XZ: original draft preparation. ZH and CD: funding acquisition. All authors have reviewed and approved the final version of the manuscript for publication.

## Funding

This study was funded by “Traditional Chinese Medicine Inheritance and Innovation Plan” of Hubei University of Chinese Medicine in 2021.

## Conflict of interest

The authors declare that the research was conducted in the absence of any commercial or financial relationships that could be construed as a potential conflict of interest.

## Publisher’s note

All claims expressed in this article are solely those of the authors and do not necessarily represent those of their affiliated organizations, or those of the publisher, the editors and the reviewers. Any product that may be evaluated in this article, or claim that may be made by its manufacturer, is not guaranteed or endorsed by the publisher.
